# Genetic Spectrum, Clinical Characteristics, and Molecular Pathogenesis of Hypertrophic Cardiomyopathy Requiring Heart Transplantation

**DOI:** 10.3390/jcdd12120499

**Published:** 2025-12-17

**Authors:** Sofiya Andreeva, Lyubov Korneva, Mariya Marusova, Yulia Sazonova, Alexandra Gudkova, Anna Streltsova, Svetlana Fetisova, Maria Simonenko, Anna Fanta, Andrei Semenov, Maria Bortsova, Lubov Mitrofanova, Olga Moiseeva, Alexandr Bobylev, Lidiya Gavrilova, Ivan Vikhlyantsev, Petr Fedotov, Anna Kostareva

**Affiliations:** 1 Almazov National Medical Research Center, 197341 Saint-Petersburg, Russia; andreeva.sofi2012@yandex.ru (S.A.); simonenko_ma@almazovcentre.ru (M.S.); semenov_ap@almazovcentre.ru (A.S.); lubamitr@yandex.ru (L.M.);; 2 Pavlov First Saint Petersburg State Medical University, 197022 Saint-Petersburg, Russia; 3 Scientific Center of Genetics and Life Sciences, Sirius University of Science and Technology, 354340 Sochi, Russia; bobylev1982@gmail.com; 4 Institute of Theoretical and Experimental Biophysics, Russian Academy of Sciences, 142290 Pushchino, Russia; ivanvikhlyantsev@gmail.com; 5 Pushchino Branch of the Federal State Budgetary Educational Institution of Higher Education “Russian Biotechnological University (BIOTECH University)”, 142290 Pushchino, Russia

**Keywords:** hypertrophic cardiomyopathy, heart transplantation, adverse remodeling, dilated phenotype, restrictive phenotype, non-sarcomeric genetic variants, titin, filamin C, truncating variants, Danon disease

## Abstract

Hypertrophic cardiomyopathy (HCM) progressing to end-stage heart failure and heart transplantation (HT) is a rare clinical scenario with an insufficiently explored genetic background. In this single-center retrospective cohort study, we aimed to characterize the genetic spectrum, variants of HCM adverse remodeling, and aspects of molecular pathogenesis of this subgroup. The study included 14 patients (9 females), among whom 10 developed a dilated/hypokinetic phenotype and 4 a restrictive phenotype. In 13 patients (93%), at least one pathogenic or likely pathogenic genetic variant was identified. Dilated remodeling/hypokinesis was associated with loss-of-function variants in *LAMP2* (3) in females, *ALPK3homo* (1), *MYH7* (1), *MYBPC3* (1), a heterozygous missense variant in *TRIM63* (1), *FLNCtv* (1), *TTNtv* (2). For the latter two, electrophoretic analysis of titin isoform composition and protein content in myocardial fragments from explanted hearts confirmed the functional significance of *TTN* gene variants. The restrictive phenotype in the adult group was associated with carriage of multiple pathogenic sarcomere gene variants: *MYL3homo* (1), *MYBPC3*+*TPM1* (1), an *MYH7* converter domain variant (1), and, in one child, with a *TNNT2* variant. This findings support HCM progressing to HT is characterized by a higher frequency of variants in non-sarcomeric genes and Danon disease compared to the general HCM cohort.

## 1. Introduction

Hypertrophic cardiomyopathy (HCM) is one of the most common inherited cardiac disorders [1]. It demonstrates pronounced phenotypic heterogeneity, encompassing variation in myocardial morphology and the degree of hypertrophy, as well as differences in the extent of interstitial and replacement fibrosis, the presence and severity of diastolic dysfunction, and incomplete penetrance [1]. Consequently, the risk of arrhythmic events and progression to heart failure varies considerably across patients, driving an ongoing search for robust high-risk markers, including those related to genetic etiology.

Adverse myocardial remodeling in HCM can evolve along two major trajectories. One is a dilated “burn-out” phase, characterized by declining contractility, chamber enlargement, and wall thinning. The other is a restrictive phenotype, marked by severe diastolic dysfunction and often accompanied by a small left-ventricular (LV) cavity and low stroke volume [2,3]. In such cases, the ejection fraction (EF) may remain preserved or only mildly reduced [2,3]. Registry data suggest that LV systolic dysfunction occurs in ~8% of HCM patients, while the restrictive phenotype accounts for ~1.3% [4,5]. According to the UNOS registry, patients with HCM represent ~2% of those undergoing heart transplantation (HT) for end-stage heart failure [6].

Although many studies support the role of genotype in determining prognosis in HCM, some evidence of inconsistency is still there [4,7,8]. This inconsistency likely reflects the heterogeneity of causal variants—acting through distinct deleterious mechanisms and with variable effect sizes. Its impact may be obscured when patients are classified simply as genotype-positive or genotype-negative, with acquired factors adding to the complexity of disease penetrance and expressivity [9]; also, major adverse outcomes remain relatively infrequent in the broader HCM population [4,6].

Therefore, we aimed to characterize the spectrum of genetic findings, clinical course, variants of adverse myocardial remodeling, and elements of molecular pathogenesis in patients with HCM who progressed to end-stage heart failure requiring heart transplantation—a patient group exhibiting one of the most severe clinical scenarios.

## 2. Materials and Methods

We analyzed patients managed between 2010 and 2025 at the Almazov National Medical Research Center (Saint Petersburg, Russia). The diagnosis of HCM as a cardiomyopathy phenotype (ESC 2023 criteria [1]) was based on imaging data, including echocardiography and cardiac magnetic resonance, and was subsequently corroborated by histological examination of explanted hearts. Patterns of myocardial remodeling were assessed, and patients were classified into dilated/hypokinetic or restrictive phenotypes. Statistical analyses and figure generation were performed using Stata 18.

Genetic testing was performed using next-generation sequencing with Sanger confirmation. The number of genes included in the panels (39, 108, or 172) varied according to the diagnostic strategy applied by the laboratory (detailed gene lists are provided in Appendix A). Testing could be carried out either before or after HT. Target enrichment was performed with SureSelect (Agilent Technologies, Santa Clara, CA, USA), and sequencing was conducted on an Illumina HiSeq platform with SBSv4 reagents (Illumina, San Diego, CA, USA). Sequence alignment, processing, and annotation were performed against the hg38 human genome reference [10]. All novel and previously reported variants with an allele frequency <0.01% were classified according to ACMG recommendations. For detailed analyses, we focused on pathogenic, likely pathogenic, and variants of uncertain significance in genes with high myocardial expression [10].

Electrophoretic separation of titin isoforms in myocardial fragments from explanted hearts [11,12], including the NT isoform described in mammalian striated muscle [13], was performed in 2.3% large-pore polyacrylamide gels reinforced with agarose, following the method of Yakupova et al. [14] with modifications. In particular, during sample preparation for SDS-PAGE, heating was limited to ≤40 °C to preserve protein integrity [15]. Titin content was quantified by densitometry relative to myosin heavy chains, an established approach for evaluating changes in high-molecular-weight titin isoforms.

## 3. Results

### 3.1. General Clinical Characteristics

**Figure 1:**
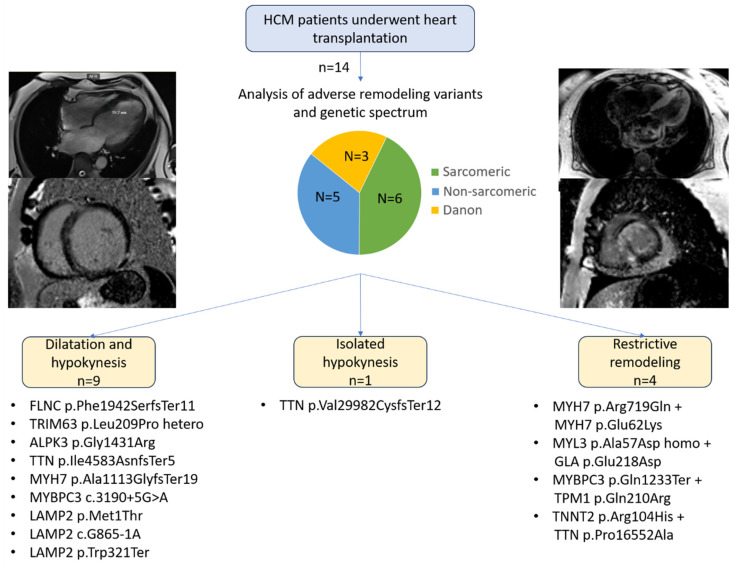
Study design and results. The central pie chart reflects the proportion of sarcomeric, non-sarcomeric variant carriers, and patients with Danon disease. MRI pictures on the left side represent dilated remodeling of patient 2 with the *TRIM63* variant, and on the right side, restrictive remodeling of patient 11 with the *MYH7* variant. HCM—hypertrophic cardiomyopathy, MRI—magnetic resonance imaging.

The study included 14 patients, 9 of them women (64%) (Table 1, Figure 1). The mean age at symptom onset was 30.6 ± 3.6 years, and the mean age at HT was 38.4 ± 3.5 years.

The mean time from diagnosis to HT was 7.8 ± 1.3 years (Table 1). A history of hypertension was present in three patients (21.4%), all diagnosed after the age of 40. Atrial fibrillation occurred in nine patients (64.3%), and all patients had documented high-grade ventricular arrhythmias. None had concomitant coronary artery disease or diabetes (Table 1).

**Figure 2:**
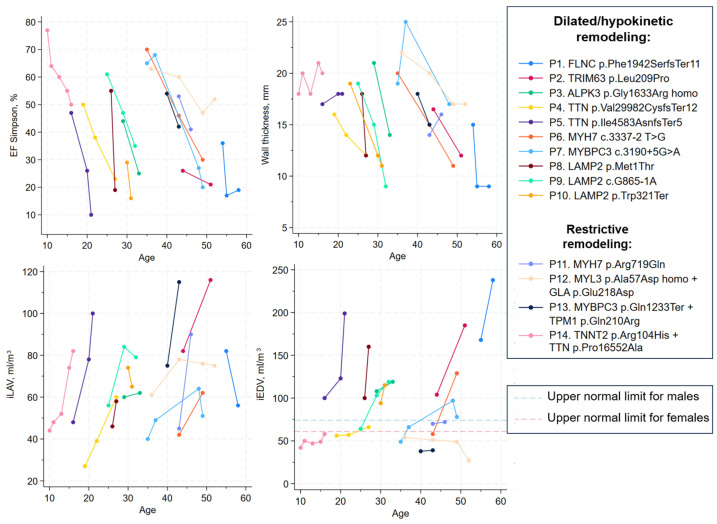
Individual patient trends of left ventricle adverse remodeling based on echocardiographic data (spaghetti plots). Patients with dilated remodeling demonstrated progressive decrease in ejection fraction, thinning of the left ventricle walls and enlargement of left ventricle (iEDV) and atrium (iLAV) over the time, while patients with restrictive remodeling had preserved or mildly reduced ejection fraction even at the terminal stage of the disease and severe atria enlargement with relatively preserved left ventricle volume (hallmark of restrictive phenotype). EF—ejection fraction; iEDV—indexed left ventricle end-diastolic volume; iLAV—indexed left atrium volume.

In nine patients (64.3%), adverse remodeling primarily manifested as dilation and hypokinesis; in one patient (7.1%), isolated hypokinesis occurred without marked dilation; in four patients (28.6%), a restrictive phenotype was observed (Figure 1). In the dilated phenotype subgroup, the mean maximal LV wall thickness decreased from 18.6 ± 0.9 mm at disease onset to 12.7 ± 0.9 mm at the terminal stage (Table 1). In the restrictive subgroup, the respective values were 18.0 ± 1.6 mm and 17.0 ± 1.1 mm (Table 1). Individual patient trends of LV adverse remodeling based on echocardiographic data are presented as spaghetti plots in Figure 2.

No patient had evidence of LV outflow tract obstruction at presentation, nor had any undergone septal myectomy (Table 1). Right ventricular (RV) hypertrophy was observed in 9 of 14 patients (64%). Increased trabeculation or criteria consistent with noncompaction myocardium were documented at certain stages of disease progression in 7 of 14 patients (50%) (Table 1).

### 3.2. Genetic Variants and Association with Clinical Course and Adverse Remodeling

Variants in sarcomere genes were identified in six patients: *TNNT2*, *MYH7* (n = 2), *MYBPC3*, *MYBPC3*+*TPM1*, and homozygous *MYL3* (Figure 1 and Table 2). Variants in non-sarcomeric or non-contractile genes were found in five patients: *ALPK3homo*, *FLNC* frameshift (n = 2), *TTN* frameshift variants (n = 2), and a *TRIM63* missense variant (Figure 1, Table 2). In three female patients, genetic testing revealed Danon disease (*LAMP2*), which had not been clinically suspected prior to testing (Figure 1 and Table 2). In 13 of 14 patients (93%), at least one variant was classified as pathogenic or likely pathogenic (Table 2).

**Figure 3:**
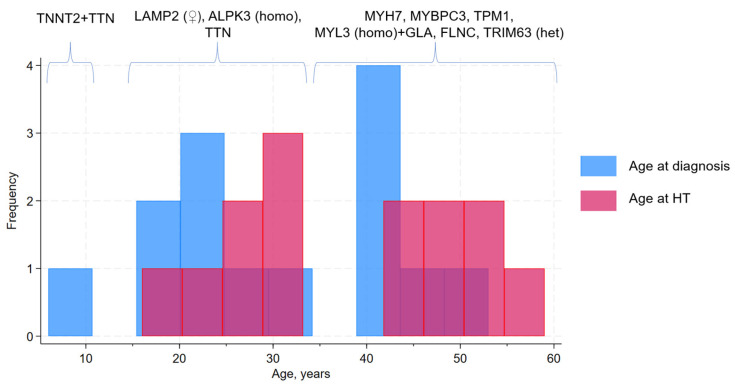
Age at diagnosis, age at heart transplantation, and underlying genes. HT—heart transplantation; het—heterozygous variants; homo—homozygous variants; ♀—female sex.

Disease onset displayed three peaks: in childhood, at 20–30 years, and after 40 years (Figure 3). Adults transplanted before 35 years included all patients with Danon disease (P.8–P.10), one patient with a homozygous missense variant in *ALPK3* (P.3), and both patients with *TTN* variants (P.4–P.5) (Table 2, Figure 3). Patients transplanted after 45 years included P.6–P.7 and P.11–P.13, who carried variants in *MYH7*, *MYL3*, *TRIM63*, and *FLNC* (Table 2, Figure 3). The pediatric subgroup was represented by P.14, who carried a likely pathogenic *TNNT2* variant in combination with a *TTN* variant of uncertain significance (Table 2, Figure 3); however, childhood-onset end-stage HCM is underrepresented in this study owing to the specifics of the pediatric HT system in Russia.

The restrictive phenotype subgroup (P.11–P.14) was enriched for carriage of multiple variants in sarcomere genes (homozygous *MYL3* and *MYBPC3* + *TPM1*) (Figure 1 and Figure 2). Of note, P.12 (female) carried homozygous *MYL3* p.Ala57Asp together with a *GLA* variant of uncertain significance linked to Fabry disease; segregation data were unavailable to clarify pathogenicity. This subgroup also included a patient with the *MYH7* converter-domain variant p.Arg719Gln.

The dilated phenotype subgroup (P.1–P.10) included all patients with Danon disease, the patient with a homozygous *ALPK3* variant, carriers of in-frame deletions in *TTN* and *FLNC*, a heterozygous *TRIM63* missense variant, and carriers of splice-site variants in *MYH7* and *MYBPC3* (Figure 1 and Figure 2, Table 2). Dilated transformation in patients with *ALPK3* and *LAMP2* variants occurred against a background of histologically verified (Dallas criteria) chronic lymphocytic, virus-negative myocarditis (per endomyocardial biopsy or explanted heart specimens). Patient 9 received immunosuppressive therapy, which did not modify the avert progression to HT. Notably, none of the women with Danon disease showed evidence of multisystem involvement (skeletal muscle, liver, or nervous system).

**Figure 4:**
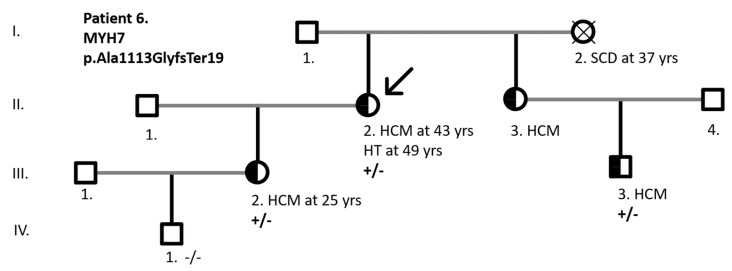
Pedigree and segregation analysis of female patient 6 with the *MYH7* p.Ala1113GlyfsTer19 variant. HCM—hypertrophic cardiomyopathy; HT—heart transplantation; SCD—sudden cardiac death. Black shading indicates the presence of the HCM phenotype, squares represent males, circles females. “+/−“ denotes heterozygous carriage of the variant; “−/−“ denotes absence of the variant.

In P.6 with the *MYH7* splice-site+frameshift variant p.Ala1113GlyfsTer19, causing the skipping of exon 27, segregation analysis enabled classification of the variant as likely pathogenic (Figure 4).

### 3.3. Titin Isoforms Electrophoretic Analysis

**Figure 5:**
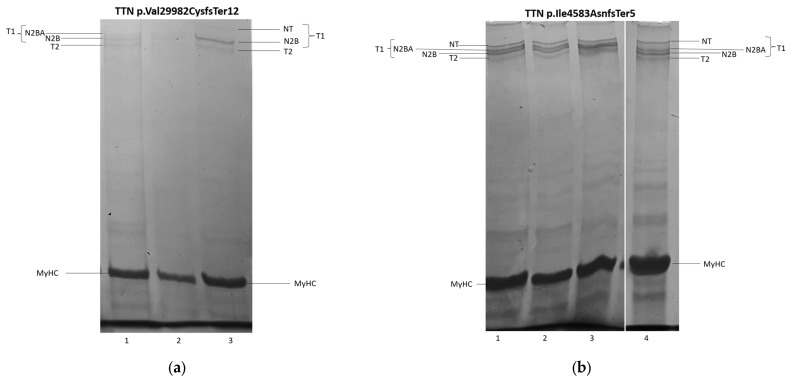
SDS–PAGE of titin in patients with truncating *TTN* variants. (**a**) Female patient 4 with p.Val29982CysfsTer12: (1) RV, (2) LV, (3) rat heart (control). There is a twofold reduction in full-length T1 molecules, a complete absence of the NT isoform, and an increased content of proteolytic T2 fragments compared with the control. (**b**) Male patient 5 with p.Ile4583AsnfsTer5: (1, 2) control (human myocardium), (3) LV, (4) RV. In both ventricles, there is an approximately twofold increase in proteolytic T2 fragments compared with control, with preserved overall content of N2BA and N2B isoforms and a reduced NT isoform in the right ventricle. Notes: LV—left ventricle; RV—right ventricle; MyHC—myosin heavy chains.

To assess the functional consequences of truncating *TTN* variants in P.4 and P.5, electrophoretic analysis of titin isoform composition and content was performed in myocardial samples from the explanted hearts (Figure 5). In P.4 (p.Val29982CysfsTer12), there was a twofold reduction in intact full-length titin-1 (T1) molecules, spanning the sarcomere from the M-line to the Z-disk (sum of NT, N2BA, and N2B isoforms)—48% in the LV and 58% in the RV compared with 100% in the control. The largest titin isoform (NT) was completely absent on the electrophoretograms of both the RV and LV. These changes indicate enhanced titin proteolysis in the patient’s heart, as further evidenced by a marked increase in the ratio of proteolytic T2 fragments to intact T1 isoforms (T2/T1 ratio 42%/58% vs. 26%/74% in control) (Figure 5a (1–3)).

In P.5 (p.Ile4583AsnfsTer5), the overall content of T1 isoforms was preserved in both ventricles; however, NT isoform abundance in the LV was reduced 1.5-fold (67% of control). In the myocardium of both ventricles, there was a more than twofold increase in the content of proteolytic T2 fragments of titin, which interact with myosin filaments in the A-band of the sarcomere. These findings suggest enhanced proteolysis of high-molecular-weight T1 isoforms and possibly increased titin turnover (Figure 5b (1–4)).

Thus, electrophoretic analysis of titin isoform composition and content demonstrated the pathogenic impact of the identified TTNtv, manifested in particular by enhanced proteolytic degradation of the protein and by a reduction in the quantitative content of its isoforms.

## 4. Discussion

In this study, we specifically examined the genetic spectrum in a cohort of HCM patients who underwent HT; to our knowledge, no prior publications have used a comparable design. Our findings may help delineate genetic subgroups at highest risk of progressing to end-stage heart failure despite contemporary guideline-directed medical therapy.

Variants in “non-sarcomeric” or “non-contractile” genes (*ALPK3*, *TTN*, *FLNC*, and *TRIM63*) accounted for 35.7% of our cohort—a proportion substantially higher than in unselected HCM populations, where non-sarcomeric gene variants account for less than 10% [21]. They are not classified as “classic” sarcomere HCM-associated genes, although some are also described as sarcomere-associated [22,23]. Importantly, variants in classic sarcomeric/contractile genes comprised 42.8%, and genotype-negative patients were virtually absent, while 21.4% of female patients were diagnosed with Danon disease (*LAMP2*). Therefore, the genotype spectrum of HCM-HT patients differs from unselected HCM cohort.

We identified patients carrying frameshift variants of *TTN* (n = 2) and *FLNC* (n = 1), resulting in truncating proteins, which are classically associated with dilated cardiomyopathy [24,25], whereas the association of *TTNtv* with HCM has been reported only sporadically [20]. Mechanisms underlying *TTNtv*-associated cardiomyopathy may include haploinsufficiency and aggregate formation due to impaired ubiquitin-mediated degradation [26]; the amyloidogenic potential of titin has also been described [27]. Previously, we reported a patient with a mixed cardiomyopathy phenotype carrying an extended deletion in *TTN*, for which bioinformatic modeling showed unfolding of protein motifs and the formation of amyloid-like structures [28]. In the patient with the *TTN* variant p.Val29982CysfsTer12, results of SDS-gel electrophoresis followed by densitometric analysis revealed reduced titin isoform content in the myocardium of the explanted heart, thereby confirming the pathogenic effect of the identified variant and suggesting a possible contribution of titin haploinsufficiency to the phenotype. In contrast, in the patient with the *TTN* variant p.Ile4583AsnfsTer5, the predominant abnormality was not enhanced proteolysis but rather accelerated titin turnover, which suggests pathological aggregation of T2 fragments and shorter titin fragments.

No descriptions of frameshift or truncating *FLNC* variants associated with HCM were identified in the available literature. Two principal mechanisms have been proposed for *FLNC*-related cardiomyopathy: protein aggregation caused by non-truncating mutations, and haploinsufficiency due to truncating mutations [29]. For HCM, protein aggregation appears to be the more likely mechanism, but this mechanism is not typical for *FLNCtv*. In this regard, it may be assumed that in patients with FLNCtv, the development of an HCM phenotype is also possible—either due to protein aggregation or through other mechanisms—or that in our patient, *FLNCtv* acted as a trigger of dilated transformation, whereas the HCM phenotype arose from other, unidentified causes, such as an non-target genetic background or the influence of polygenic determinants in an older patient with a history of hypertension [9]. Confirmation of the first hypothesis would require advanced proteomic and structural studies. The second hypothesis is consistent with current evidence that HCM may develop as a polygenic disorder with additional contributions from acquired factors [9,30].

The latter assumption can also be relevant for the patient with a *TRIM63* variant in a heterozygous state. For *TRIM63*, an autosomal-recessive inheritance model in HCM is now considered highly probable [14]. However, we cannot exclude a modifying contribution of heterozygous missense *TRIM63* variants in patients with poorly controlled hypertension, as in our case.

In female patients with Danon disease, beyond the well-described isolated cardiac involvement without significant multisystem manifestations, we emphasize the possible role of myocarditis in accelerating fibrosis and driving heart failure, potentially reflecting an autoimmune response to primary genetic injury. Such a mechanism may also be relevant in other genetic cardiomyopathies. Taking into account the histological signs of myocarditis in the *ALPK3* patient as well, this mechanism can be suggested as an important factor triggering malignant remodeling.

For *ALPK3*, homozygous loss-of-function variants have been linked to severe autosomal-recessive HCM with childhood onset [22]. More recently, heterozygous loss-of-function variants were shown to underlie autosomal-dominant adult-onset HCM with reduced penetrance, resembling genotype-negative disease [22,31]. In this study, we report a case of HCM associated with a homozygous *ALPK3* missense variant identified at a young age, which may indicate a role of missense variants in the development of autosomal-recessive *ALPK3* cardiomyopathy. The clinical course and genetic data of this patient have been described in detail previously [15].

Special attention should be paid to variants in classic HCM-associated sarcomeric genes and their combinations. One of our patients carried a splice-site variant in *MYH7* leading to exon 27 skipping, which implies haploinsufficiency. This mechanism is atypical for *MYH7*-related HCM but more characteristic of dilated cardiomyopathy and LV noncompaction [32]. Another patient carried *MYH7* p.Arg719Gln and p.Glu62Lys, located in the converter and SH3 domains, respectively. Compound heterozygosity and digenic inheritance are well-known to be connected with a more malignant and penetrant HCM phenotype [21,33]. Variants in the *MYH7* converter domain (amino acids 709–777) are well known to be associated with adverse prognosis. The p.Arg719Gln variant has also been previously reported as pathogenic, characterized by high penetrance, increased risk of sudden cardiac death, and absence of intraventricular obstruction [18,34]. Notably, atrial enlargement and atrial fibrillation have been described in a number of patients [35], features consistent with the restrictive phenotype observed in our patient. Nevertheless, it cannot be excluded that the second variant, p.Glu62Lys, also contributed to the development of restrictive hemodynamics.

For the *MYL3* gene, usually linked to autosomal-dominant HCM, an autosomal-recessive form has recently been described [19] for the variant p.Ala57Asp, but without restrictive features. This variant was also described as a variant with an intermediate effect in the paper of Hernandez SG et al. [21]. In this context, in our patient, the restrictive phenotype may have been influenced by concomitant carriage of a *GLA* variant of uncertain significance, but segregation analysis was not available.

The limitations of our study include its retrospective cohort design, the use of different gene panels across patients (Table 1), and restriction to individuals who underwent HT rather than all patients with end-stage heart failure. Also, childhood-onset end-stage HCM is underrepresented in our cohort, as pediatric organ donation is not permitted in Russia, and size incompatibility often precludes transplantation from adult donors. This subgroup warrants focused study in the future.

## 5. Conclusions

The genetic basis of HCM progressing to HT is heterogeneous. In comparison with unselected HCM cohorts, our series was markedly enriched for non-sarcomeric variants. Among sarcomeric genes, carriage of multiple pathogenic variants (within a single gene or across genes) and *MYH7* converter-domain variants appear to predispose to restrictive remodeling, whereas splice-site variants in *MYH7* and *MYBPC3* may lead to haploinsufficiency and dilated transformation. Danon disease accounted for a substantial proportion of cases, and its storage-disease nature may remain unrecognized until genetic testing, particularly in women. Potentially high-risk non-sarcomeric variants include homozygous *ALPK3*. Finally, truncating variants in sarcomere-associated genes not typically linked to HCM, such as *TTN* and *FLNC*, may also contribute to HCM or its dilated phase, likely through haploinsufficiency or protein aggregation.

## Figures and Tables

**Table 1:** Clinical characteristics of 14 HCM patients with adverse LV remodeling (dilated or restrictive), requiring heart transplantation. AF—atrial fibrillation, EF—ejection fraction, HCM—hypertrophic cardiomyopathy, HT—heart transplantation, iLAV—indexed left atrium volume, iEDV—indexed end-diastolic volume, LV—left ventricle, NT-proBNP—N-terminal pro-brain natriuretic peptide, RV—right ventricle.

Mean (SD)		Dilated Remodeling (n = 10)	Restrictive Remodeling (n = 4)	Overall (n = 14)
EF, %	at debut	48.3 (4.7)	61.8 (5.6)	52.1 (4.0)
	at terminal stage	22.3 (2.2)	44.5 (2.)	28.6 (3.2)
Wall thickness, mm	at debut	18.6 (0.9)	18 (1.60)	18.5 (0.8)
	at terminal stage	12.7 (0.9)	17 (1.1)	13.9 (0.9)
iEDV, mL/m^3^	at debut	90.1 (11.2)	51.0 (7.2)	78.9 (9.5)
	at terminal stage	140.8 (17.1)	49.0 (10.0)	114.6 (16.)
iLAV, mL/m^3^	at debut	55.7 (6.0)	56.3 (7.4)	55.9 (4.6)
	at terminal stage	70.9 (6.7)	90.8 (8.6)	76.6 (5.8)
RV hypertrophy		5 (50%)	4 (100%)	9 (64%)
LV hypertrabeculation		6 (60%)	1 (25%)	7 (50%)
Female sex		6 (60%)	3 (75)	9 (64%)
AF		6 (60%)	3 (75%)	9 (64%)
Hypertension		3 (30%)	0	3 (21%)
Septal myoectomy		0	0	0
Coronary artery disease		0	0	0
Diabetes		0	0	0
Liver fibrosis		0	2 (50%)	2 (14%)
Precapillary pulmonary hypertension		2 (20%)	2 (50%)	4 (29%)
NT-proBNP, ng/mL		6509.2 (1682.0)	4444.5 (741.0)	5873.9 (1193.2)
Time from debut to HT		6.9 (0.8)	10 (4.2)	7.8 (1.3)
Age at debut		31.1 (4.2)	29.5 (8.3)	30.6 (3.6)
Age at HT		38.0 (4.1)	39.5 (8.0)	38.4 (3.5)

**Table 2:** Characteristics of the genetic variants identified in the study patients. ACMG—American College of Medical Genetics; EF—ejection fraction; F—female; HF—heart failure; iEDV—indexed left ventricle end-diastolic volume; IVS—interventricular septum; LP—likely pathogenic; M—male; MAF—minor allele frequency; P—pathogenic; RCM—restrictive cardiomyopathy; SCD—sudden cardiac death; VUS—variant of uncertain significance.

Patient, Sex	Gene	GRCh38 Position and Nomenclature	Variant Type	Rs, MAF%, Literature Reference	Zygosity, ClinGen Evaluation	ACMG Class	Gene Panel	Age at Diagnosis	Age at HT	Initial Echo	Family History
**1 M**	FLNC	Chr7:128851609, NM_001458.5:c.5823delC; p.Phe1942SerfsTer11	Frame-shift deletion	-	Hetero, definitive	LP	39	53	59	EF—36%, IVS—15 mm, iEDV—168 mL/m^3^	Father—SCD at 50 yrs
**2 M**	TRIM63	Chr1: 26058595, NM_032588.4:c.T626C; p.Leu209Pro	Missense	rs1553145730 0.00007	Hetero, disputed	VUS	39	44	52	EF—26%, IVS—17 mm, iEDV—104 mL/m^3^	Father—fatal stroke at 50, mother—HF at 45 yrs
**3 F**	ALPK3	Chr15: 8486279, NM_020778.5:c.G4291GA; p.Gly1431Arg	Missense	rs750258262 0.001 [10]	**Homo,** definitive	LP	172	23	33	EF—44%, IVS—21 mm, iEDV—108 mL/m^3^	Father—SCD at 40 yrs
**4 F**	TTN	Chr2: 178552954, NM_001267550.2:c.89943_89946del; p.Val29982CysfsTer12	Frame-shift deletion	-	Hetero, limited	LP	172	17	27	EF—50%, IVS—16 mm, iEDV—56 mL/m^3^	Unremarkable
		Chr2: 178616815, NM_001267550.2:c.G48074A; p.Ser16025Asn	Missense	rs727504720 0.00007	Hetero, limited	VUS					
**5 M**	TTN	Chr2: 178748649: NM_133379.5:c.13748_13751del; p.Ile4583AsnfsTer5	Frame-shift deletion	rs1460696675 0.002	Hetero, limited	LP	172	16	21	EF—47%, IVS—17 mm, iEDV—100 mL/m^3^	Unremarkable
**6 F**	MYH7	Chr14: 23420234, NM_000257.4:c.3337dup; p. Ala1113GlyfsTer19	Splice-region+ frameshift		Hetero, definitive	LP	108	43	49	EF—70%, IVS—20 mm, iEDV—58 mL/m^3^	Mother—SCD at 37, HCM in daughter, sister, and nephew
**7 M**	MYBPC3	Chr11: 47333552, NM_000256.3:c.G3190+5A	Splice-region	rs587782958 0.0006 [16]	Hetero, definitive	P	39	35	50	EF—65%, IVS—19 mm, iEDV—49 mL/m^3^	Father—SCD at 56
**8 F**	LAMP2	ChrX: 120469168, NM_002294.3:c.T2C; p.Met1Thr	Start-loss	-	Hetero, definitive	LP	39	26	28	EF—55%, IVS—18 mm, iEDV—100 mL/m^3^	Unremarkable
**9 F**	LAMP2	ChrX: 120442663, NM_002294.3:c.G865-1C -	Splice-region	rs397516752	Hetero, definitive	P	172	23	32	EF—61%, IVS—19 mm, iEDV—64 mL/m^3^	Grandmother—SCD at 53
**10 F**	LAMP2	ChrX: 120441861, NM_002294.3:c.G962GA; p.Trp321Ter	Nonsense	rs1060502306 [17]	Hetero, definitive	P	39	23	30	EF—29%, IVS—19 mm, iEDV—94 mL/m^3^	Mother—HF, SCD at 60
**11 F**	MYH7	Chr14: 23425970, NM_000257.4:c.G2156GA; p.Arg719Gln	Missense	rs121913641 [18]	Hetero, definitive	P	39	42	46	EF—53%, IVS—16 mm, iEDV—70 mL/m^3^	Father—SCD at 55
		Chr14: 23433549: NM_000257.4:c.G184A; p.Glu62Lys	Missense	rs727504416 0.0005	Hetero, definitive	VUS					
**12 F**	MYL3	Chr3: 46860813:NM_000258.3:c.C170A; p.Ala57Asp	Missense	rs139794067 0.01 [19]	**Homo,** definitive	LP	39	30	52	EF—63%, IVS—22 mm, iEDV—54 mL/m^3^	Unremarkable
	GLA	ChrX: 101398932, NM_000169.3: c.A654T; p.Glu218Asp	Missense	-	Hetero, definitive	VUS					
**13 F**	MYBPC3	Chr11: 47332189: NM_000256.3:c.C3697T; p.Gln1233Ter	Nonsense	rs397516037 0.0008 [20]	Hetero, definitive	P	172	40	44	EF—54%, IVS—18 mm, iEDV—38 mL/m^3^	Mother—HF, death at 40 yrs, autopsy diagnosis—RCM. Sister—SCD at 12 yrs.
	TPM1	Chr15:63061778: NM_001018005.2: c.A629G; p.Gln210Arg	Missense	rs777139450	Hetero, definitive	LP					
**14 M**	TNNT2	Chr1:201365291: NM_001276345.2:c.G311A; p.Arg104His	Missense	rs397516457 [9]	Hetero, definitive	P	172	6	16	EF—77%, IVS—18 mm, iEDV—42 mL/m^3^	Unremarkable
	TTN	Chr2:178613067: NM_001267550.2:c.C49654G; p.Pro16552Ala	Missense	-	Hetero, limited	VUS					

## Data Availability

Data is contained within the article.

## Abbreviations

The following abbreviations are used in this manuscript:

ACMG	American College of Medical Genetics
HCM	Hypertrophic cardiomyopathy
HT	Heart transplantation
LV	Left ventricle
RV	Right ventricle

## Appendix A

*Targeted 172-gene panel:*

*ABCC9*, *ACADVL*, *ACTA1*, *ACTC1*, *ACTN2*, *ACVR2B*, *AGK*, *AKAP9*, *ALPK3*, *ANK2*, *ANKRD1*, *ANO5*, *BAG3*, *BRAF*, *CACNA1C*, *CACNA2D1*, *CACNB2*, *CALM1*, *CALM2*, *CALM3*, *CALR3*, *CASQ2*, *CAV3*, *CBL*, *CDH2*, *CMYA5*, *CRELD1*, *CRYAB*, *CSRP3*, *CTNNA3*, *DES*, *DMD*, *DMPK*, *DNAAF1*, *DNAAF3*, *DPP6*, *DSC2*, *DSG2*, *DSP*, *DTNA*, *DYSF*, *EMD*, *EYA4*, *FHL1*, *FHL2*, *FHOD3*, *FKRP*, *FKTN*, *FLNA*, *FLNC*, *FXN*, *GAA*, *GATA4*, *GATA5*, *GATA6*, *GATAD1*, *GDF1*, *GJA5*, *GLA*, *GPD1L*, *HAND1*, *HCN4*, *HFE*, *HRAS*, *ILK*, *ISPD*, *JPH2*, *JUP*, *KCNA5*, *KCND3*, *KCNE1*, *KCNE2*, *KCNE3*, *KCNE5*, *KCNH2*, *CNJ2*, *KCNJ5*, *KCNJ8*, *KCNQ1*, *KRAS*, *LAMA4*, *LAMP2*, *LDB3*, *LEFTY2*, *LMNA*, *LMOD3*, *LRRC10*, *LZTR1*, *MAP2K1*, *MAP2K2*, *MIB1*, *MMP21*, *MRA*, *MYBPC3*, *MYBPHL*, *MYH6*, *MYH7*, *MYL2*, *MYL3*, *MYL4*, *MYLK2*, *MYOF*, *MYOM1*, *MYOT*, *MYOZ2*, *MYPN*, *NEBL*, *NEXN*, *NF1*, *NKX2-5*, *NKX2-6*, *NPPA*, *NRAS*, *NUP155*, *PDLIM3*, *PKD1L1*, *PKP2*, *PLEC*, *PLEKHM2*, *PLN*, *PPA2*, *PPP1CB*, *PRDM16*, *PRKAG2*, *PSEN1*, *PSEN2*, *PTPN11*, *RAF1*, *RANGRF*, *RBM20*, *RIT1*, *RRAS*, *RYR2*, *SALL4*, *SCN10A*, *SCN1B*, *SCN2B*, *SCN3B*, *SCN4B*, *SCN5A*, *SCNN1G*, *SDHA*, *SGCD*, *SHOC2*, *SLMAP*, *SNTA1*, *SOS1*, *SOS2*, *SPEG*, *SPRED1*, *SYNE1*, *SYNM*, *SYNPO2L*, *TAZ*, *TBX20*, *TBX5*, *TCAP*, *TECRL*, *TGFB3*, *TMEM43*, *TMPO*, *TNNC1*, *TNNI3*, *TNNI3K*, *TNNT2*, *TPM1*, *TRDN*, *TRPM4*, *TTN*, *TTR*, *VCL*, *ZIC3*.

*Targeted 108-gene panel:*

*ABCC9*, *ACTC1*, *ACTN2*, *AKAP9*, *ANK2*, *ANKRD1*, *BAG3*, *BRAF*, *CACNA1C*, *CACNA2D1*, *CACNB2*, *CALM1*, *CALR3*, *CASQ2*, *CAV3*, *CBL*, *CRYAB*, *CSRP3*, *DES*, *DMD*, *DMPK*, *DSC2*, *DSG2*, *DSP*, *DTNA*, *EMD*, *EYA4*, *FHL1*, *FHL2*, *FKTN*, *FXN*, *GAA*, *GLA*, *GPD1L*, *HCN4*, *HRAS*, *ILK*, *JPH2*, *JUP*, *KCND3*, *KCNE1*, *KCNE1L*, *KCNE2*, *KCNE3*, *KCNH2*, *KCNJ2*, *KCNJ5*, *KCNJ8*, *KCNQ1*, *KRAS*, *LAMA4*, *LAMP2*, *LDB3*, *LMNA*, *MAP2K1*, *AP2K2*, *MRPL3*, *MYBPC3*, *MYH6*, *MYH7*, *MYL2*, *MYL3*, *MYLK2*, *MYOM1*, *MYOZ2*, *MYPN*, *NEBL*, *NEXN*, *NF1*, *NOS1AP*, *NRAS*, *PDLIM3*, *PKP2*, *PLN*, *PRKAG2*, *SEN1*, *PSEN2*, *PTPN11*, *RAF1*, *RANGRF*, *RBM20*, *RYR2*, *SCN1B*, *SCN3B*, *SCN4B*, *SCN5A*, *SCO2*, *SDHA*, *SGCD*, *SHOC2*, *SLC25A3*, *SLMAP*, *SNTA1*, *SOS1*, *SPRED1*, *TAZ*, *TCAP*, *TGFB3*, *TMEM43*, *TMPO*, *TNNC1*, *TNNI3*, *TNNT2*, *TPM1*, *TRDN*, *TRPM4*, *TTN*, *VCL*.

*Targeted 39-gene panel:*

*ACTC1*, *ACTN2*, *ALPK3*, *BAG3*, *BRAF*, *CBL*, *CSRP3*, *FHL2*, *FHOD3*, *FLNC*, *FXN*, *GLA*, *HRAS*, *KRAS*, *LAMP2*, *LDB3*, *LZTR1*, *MAP2K1*, *MAP2K2*, *MYBPC3*, *MYH7*, *MYL2*, *MYL3*, *MYLK2*, *MYOM1*, *MYPN*, *NF1*, *NRAS*, *PRKAG2*, *PTPN11*, *RAF1*, *SHOC2*, *SOS1*, *TNNC1*, *TNNI3*, *TNNT2*, *TPM1*, *TRIM63*, *TTR*.
